# *SiMYBS3*, Encoding a *Setaria italica* Heterosis-Related MYB Transcription Factor, Confers Drought Tolerance in *Arabidopsis*

**DOI:** 10.3390/ijms24065418

**Published:** 2023-03-12

**Authors:** Xin Liu, Shuai Zhang, Mengbo Sun, Yurong Guo, Shaoxing Zhao, Xutao Zhou, Xionghui Bai, Keli Dai, Huixia Li, Xiangyang Yuan, Weiping Shi, Pingyi Guo, Jie Guo

**Affiliations:** 1 College of Agronomy, Shanxi Agricultural University, Jinzhong 030801, China; 2 Millet Research Institute, Shanxi Agricultural University, Changzhi 046011, China

**Keywords:** hybrid vigor, foxtail millet, transcriptomics, transgenic *Arabidopsis*, drought

## Abstract

Drought is a major limiting factor affecting grain production. Drought-tolerant crop varieties are required to ensure future grain production. Here, 5597 DEGs were identified using transcriptome data before and after drought stress in foxtail millet (*Setaria italica*) hybrid *Zhangza 19* and its parents. A total of 607 drought-tolerant genes were screened through WGCNA, and 286 heterotic genes were screened according to the expression level. Among them, 18 genes overlapped. One gene, *Seita.9G321800,* encoded MYBS3 transcription factor and showed upregulated expression after drought stress. It is highly homologous with MYBS3 in maize, rice, and sorghum and was named *SiMYBS3*. Subcellular localization analysis showed that the SiMYBS3 protein was located in the nucleus and cytoplasm, and transactivation assay showed SiMYBS3 had transcriptional activation activity in yeast cells. Overexpression of *SiMYBS3* in *Arabidopsis thaliana* conferred drought tolerance, insensitivity to ABA, and earlier flowering. Our results demonstrate that *SiMYBS3* is a drought-related heterotic gene and it can be used for enhancing drought resistance in agricultural crop breeding.

## 1. Introduction

Heterosis is a phenomenon by which the F_1_ generation outperforms its parents in specific aspects, such as yield, quality, and environmental adaptation [1,2]. Heterosis has been successfully applied in agricultural production. Current hypotheses explaining heterosis including dominance, complementation, overdominance, and epistasis cannot fully explain the underlying mechanism [3]. The genetic basis of heterosis has been analyzed from a range of perspectives and various differentially expressed genes (DEGs) show potential influence on heterosis [4,5,6].

Transcription factors (TFs) are potential regulators of DEGs [7], and they also play important roles in heterosis formation [8,9,10]. These include myeloblastosis (MYB) genes, ethylene-responsive element binding factors (ERF), basic leucine zipper (bZIP), and basic helix-loop-helix (bHLH). MYB TFs, the largest TF family in plants, have been implicated in hybrid superiority. For example, *csa,* a rice mutant that encodes a MYB TF, confers male sterility in certain genotypes grown under short days, but normal fertility under long-day conditions [11]. A MYB TF in cotton plays a similar rule [12]. In sorghum, gene *Dw7a*, encoding a MYB TF, has been cloned and shows potential in hybrid breeding [13]. Likewise, *ZmMYBL1* in maize was considered to be related to hybrid dominance [14]. MYB TF genes also function in response to abiotic stresses. For example, *VyMYB24* in *Vitis yanshanesis* caused dwarfing and conferred plant drought resistance [15]. Overexpression of *GhMYB36* conferred drought tolerance in cotton and other plant species [16]. Overexpression of *PtoMYB142* in poplar significantly enhanced drought resistance [17]. Overexpression of *MbMYB108* from *Malus baccata* improved cold and drought resistance in *Arabidopsis* [18]. Overexpression of *OsMYBS3* improved cold tolerance in rice [19]. All these reports suggest that MYB TF family genes from different plant species have important roles in drought response. Therefore, are there some TFs that play an important role in the formation of drought-resistant traits in hybrids with significant drought-resistant phenotypes?

Foxtail millet (*Setaria italica*) is a model plant for stress tolerance studies in C_4_ crop species [20,21]. It is barren tolerant, drought tolerant, and has a small genome and short growth cycle [21,22,23,24]. “*Zhangza*” is a series name for a group of hybrids with higher yield, superior water conservation, and greater drought tolerance than conventional varieties cultivated in northern China [25]. As the most drought-resistant variety in the *Zhangza* series, *Zhangza 19* was tested in an extremely arid region of Dunhuang Gansu province. The successful trial of *Zhangza 19* demonstrated the advantage of hybrids under drought-prone conditions. However, the potential application of drought-tolerant genes to improve drought tolerance in foxtail millet is barely reported. It is of great significance to successfully exploit the drought-tolerant genes of foxtail millet by using *Zhangza 19*. At present, no in-depth analysis has been conducted on the formation of drought resistance traits in *Zhangza 19*. The exploration of drought resistance genes in *Zhangza 19*, especially the genes encoding transcription factors, is of great significance for the study of drought response mechanisms and heterosis.

The objective of the present study was to identify drought-resistant heterosis-related genes by means of a transcriptome analysis of *Zhangza 19* and its parents before and after drought stress. We constructed a co-expression gene network using WGCNA to identify genes in *Zhangza 19* associated with drought resistance and heterosis. One candidate gene, *SiMYBS3,* was cloned and transformed into *Arabidopsis* to validate its functions under drought stress. Our results provide experimental evidence for further mining and utilization of genes for drought tolerance.

## 2. Results

### 2.1. Identification of Drought-Resistant and Heterotic Genes

**Figure 1:**
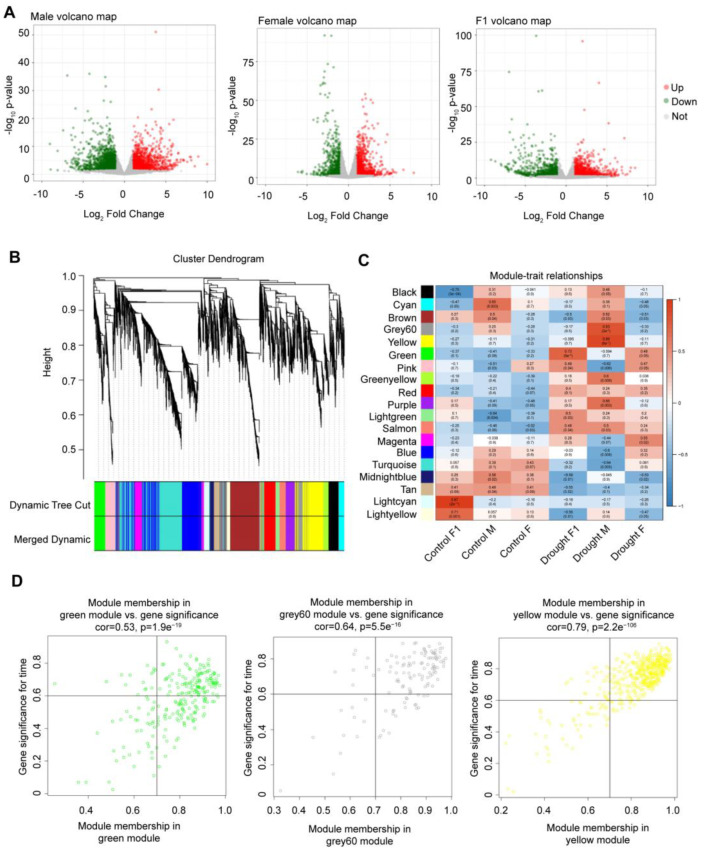
Screening of candidate genes for drought stress. (**A**) Volcano maps of F1, male, and female parents. Green and red represent downregulated genes and upregulated genes, respectively. (**B**) Hierarchical clustering diagram according to weighted gene co-expression network analysis modules. (**C**) Module–feature relationship. (**D**) Genes with high correlation coefficients in key modules (gene significance time > 0.6, module membership > 0.7).

Analysis of DEGs in *Zhangza 19* and its parents before and after drought stress identified 2283 DEGs in the hybrid (F_1_ generation), including 1212 upregulated and 1071 downregulated; 3323 in the male parent, comprising 1392 upregulated and 1931 downregulated; and 1808 in the female parent, comprising 1005 upregulated and 803 downregulated (Figure 1A). In total, 5597 DEGs were identified in all 3 groups; WGCNA identified 19 gene modules (Figure 1B), 3 of which (green, Grey60, and yellow) were significantly associated with drought resistance in the parents and F_1_ generation (Figure 1C). Among the three modules, 607 genes (including 120, 88, and 399 genes related to drought stress with significance time > 0.6 and module membership > 0.7) were identified and used for further analysis (Figure 1D).

**Figure 2:**
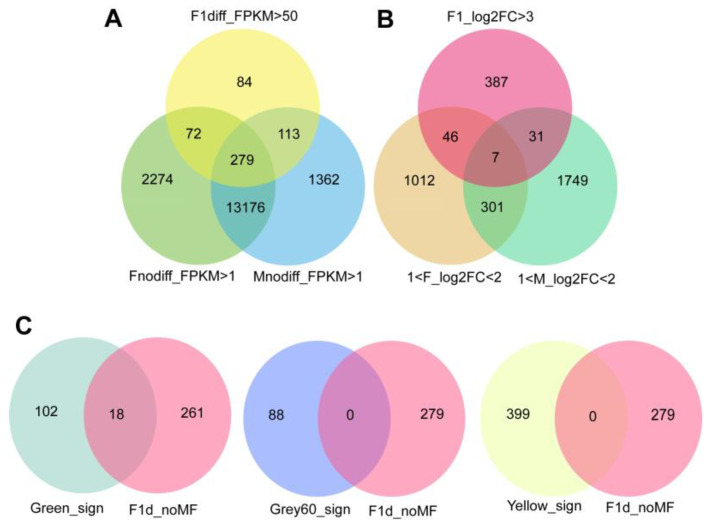
Screening candidate genes for heterosis. (**A**) Genes were expressed in the parents, but more were expressed in the hybrid. (**B**) DEGs in the parents, with increased differential fold in the hybrid. (**C**) Genes associated with both drought stress and heterosis.

Heterosis is partly due to the fact that some genes perform gene functions in parents without differential expression but are upregulated in the F_1_ generation; there were 279 genes meeting these criteria (Figure 2A). Heterosis also comes partly from the differential expression of some genes in the parents and differential multiple increases in the F_1_ generation; seven genes met these conditions (Figure 2B). These 286 genes were considered heterosis-related and used for further analysis.

A total of 18 genes were commonly identified among these 607 drought-related and 286 heterosis-related genes (Figure 2C and Supplementary Materials, Table S2).

### 2.2. Analysis of Drought-Resistant and Heterotic Genes

**Figure 3:**
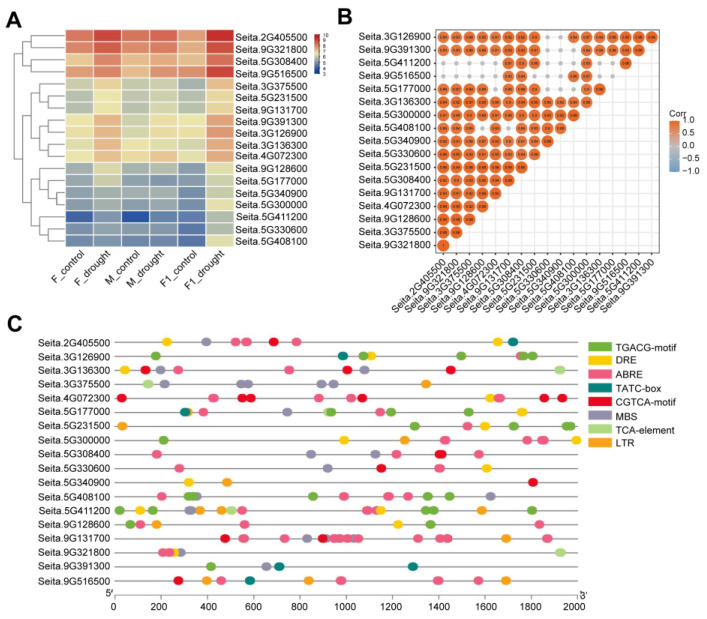
Analysis of drought resistance and heterotic genes. (**A**) Expression patterns of 18 candidate genes. (**B**) Correlation analysis of the candidate genes. (**C**) Promoter cis-regulatory element analysis of the candidate genes.

After drought stress, the 18 drought-resistant and heterosis-related genes were upregulated and highly expressed in *Zhangza 19*, compared with their parents, with *Seita.2G405500*, *Seita.9G321800*, *Seita.5G308400*, and *Seita.9G516500* having the highest expression levels (Figure 3A). Further confirmation of the correlation between these 18 genes (in Figure 3B, grey dots indicate that the genes are not correlated with each other, and the size of the values represents the size of the correlation) showed that 9 genes (*Seita.5G300000*, *Seita.5G308400*, *Seita.5G231500*, *Seita.9G131700*, *Seita.2G405500*, *Seita.9G321800*, *Seita.3G126900*, *Seita.3G136300*, and *Seita.3G375500*) were more strongly correlated with each other. *Cis*-acting element analysis of the upstream 2 kb genomic DNA sequences of these 18 genes revealed that 9 of them (*Seita.2G405500*, *Seita.3G136300*, *Seita.5G17700*, *Seita.5G308400*, *Seita.5G330600*, *Seita.5G408100*, *Seita.5G411200*, *Seita.9G131700*, and *Seita.9G321800*) have drought-responsive elements ABRE and MBS in the promoter regions (Figure 3C). *Seita.2G405500*, *Seita.9G321800*, and *Seita.5G308400* were highly expressed after drought stress and had ABRE elements; they were therefore selected as candidate genes for further analysis.

### 2.3. Phylogenetic Relationship, Expression Patterns, Subcellular Localization, and Transactivation Activity Analysis of SiMYBS3

**Figure 4:**
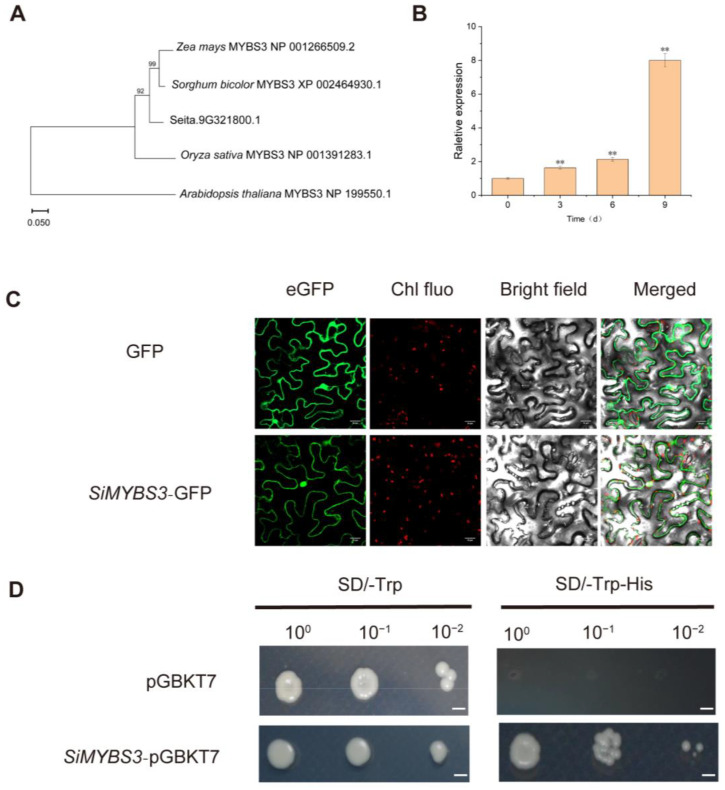
Phylogenetic relationship, expression patterns, subcellular localization, and transactivation assay of SiMYBS3. (**A**) Phylogenetic relationship of SiMYBS3 and other plant MYBS3 members. The accession numbers for different plants are: NP_001391283.1 (*Oryza sativa* MYBS3), NP_001266509.2 (*Zea mays* MYBS3), XP_002464930.1 *(Sorghum bicolor* MYBS3), and NP_199550.1 (*Arabidopsis thaliana* MYBS3). The numbers at the nodes indicate the bootstrap values. (**B**) Expression levels of *SiMYBS3* under drought stress at different time points. (**C**) Subcellular localization of SiMYBS3 in tobacco leaf cells. The fusion construct (*SiMYBS3*::GFP) was transiently expressed in tobacco epidermal cells. Empty vector (GFP) was used as control. Bar, 20 μm. (**D**) Transactivation assay of SiMYBS3. Transformed yeast cells were screened on SD/-Trp and SD/-Trp-His media. Bar, 0.2 cm. The histogram represents mean ± SD of three biological replicates. Student’s *t*-tests: ** *p* ≤ 0.01.

Among the three candidate genes, *Seita.9G321800* encoded an MYB TF family member and showed high similarity to maize, rice, and sorghum *MYBS3* genes (Figure 4A). As the rice *MYBS3* gene was known to be associated with abiotic stress, we chose *Seita.9G321800* as a candidate gene for further research and named it *SiMYBS3*.

Expression levels of *SiMYBS3* detected under drought stress treatments showed that its expression was significantly activated by drought; *SiMYBS3* expression increased with time under drought stress (Figure 4B).

SiMYBS3 fusion protein signals confirmed that the SiMYBS3 protein was in the nucleus and cytoplasm (Figure 4C). Transactivation assays showed that the yeast cells carrying the pGBKT7-SiMYBS3 and pGBKT7 plasmids grew normally on SD/-Trp medium, whereas only pGBKT7-SiMYBS3 grew normally on SD/-Trp-His medium (Figure 4D), indicating that SiMYBS3 has transcriptional activation activity.

### 2.4. Overexpression of SiMYBS3 in Arabidopsis Led to Improved Drought Tolerance

**Figure 5:**
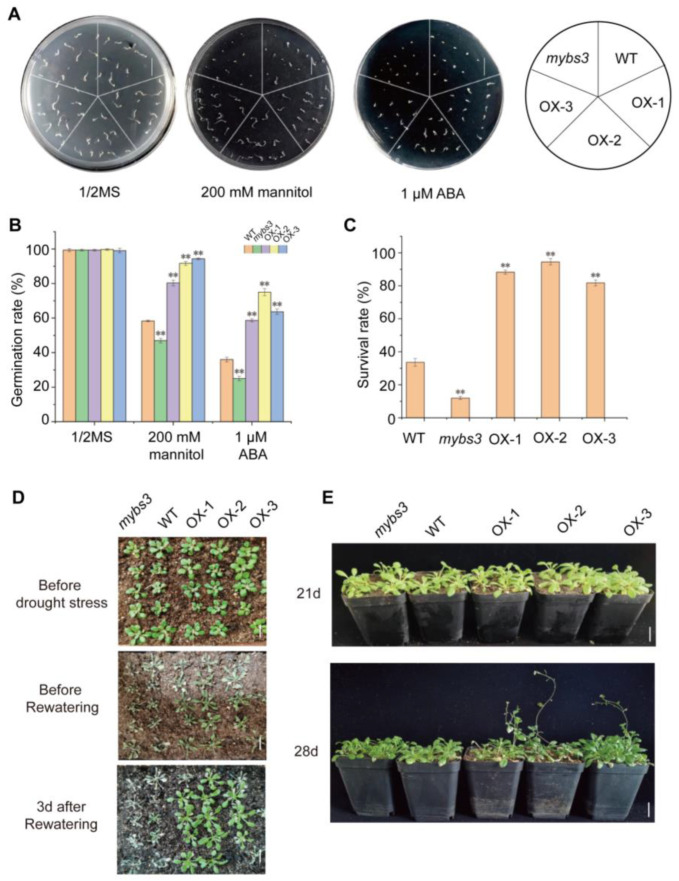
Phenotypic analysis of overexpression of *SiMYBS3* in *Arabidopsis*. (**A**) Phenotypes of *SiMYBS3* overexpression in *Arabidopsis* on ½ MS solid medium, ½ MS containing 200 mM mannitol, and ½ MS containing 1 µM ABA. Bar, 1 cm. (**B**) Germination rates of *Arabidopsis* genotypes on ½ MS with or without mannitol and ABA. (**C**) Survival rates of *Arabidopsis* genotypes after drought stress. (**D**) Phenotypes of *SiMYBS3* transgenic lines, mybs3 mutant, and WT under drought stress. Bar, 1 cm. (**E**) Phenotypes of *Arabidopsis* genotypes at 21 d and 28 d after transplanting into soil. Bar, 2 cm. The histogram represents mean ± SD of three biological replicates. Student’s *t*-tests: ** *p* ≤ 0.01.

Seeds of wild-type *Col-0*, *SiMYBS3*-overexpression transgenic lines (OX-1, -2, and -3), and *Arabidopsis* T-DNA insertion mutant *mybs3* were spotted on 1/2MS medium with and without 200 mM mannitol and 1 μM ABA to investigate the function of the *SiMYBS3* gene in drought tolerance. There was no difference in germination potential between the lines on 1/2MS medium at the 4th day after sowing (Figure 5A), whereas seed germination potential descended in the order: 3 overexpression lines > WT > *mybs3* mutant on 1/2MS medium containing 200 mM mannitol and 1 μM ABA (Figure 5A). Germination rates recorded on day 7 (Figure 5B) indicated that the *SiMYBS3*-overexpression lines had significantly higher germination rates than the WT and *mybs3* mutant on 1/2MS media containing mannitol and ABA.

We further confirmed the function of *SiMYBS3* in response to natural drought stress. The survival rates of the transgenic lines (81.7–94.5%) under drought conditions were significantly higher than that of WT (33.6%), and survival of the WT was significantly higher than that of the *mybs3* mutant (11.9%) (Figure 5C,D). The transgenic lines flowered earlier than the WT and *mybs3* mutant (Figure 5E).

## 3. Discussion

### 3.1. RNA-Seq Can Be Used to Mine Drought Resistance and Heterotic Genes

RNA-seq can be used for genetic analysis of heterosis [26]. Heterosis may be caused by DEGs between hybrids and their parents [27,28,29]. Here, DEGs in *zhangza19* and its parents were identified based on RNA-seq data. There were five types of DEGs between hybrids and parents: co-expression (M-F-F_1_), parent-specific expression (M/F), hybrid-specific expression (F_1_), single-parent silent expression (M-F_1_/F-F_1_), and hybrid-silent expression (M-F). In contrast to the other four types, the differential expression of the M-F-F_1_ gene is reflected in the difference in expression amount, which is considered the main form of differential gene expression and an important source of heterosis [30]. In this study, 286 M-F-F_1_-type genes were related to heterosis. Among them, 18 were upregulated only in F_1_ generation after drought stress and 7 differentially expressed in both parents and F_1_ generation showed no clear evidence of action in response to drought stress (Supplementary Materials, Figure S1). Therefore, it can be inferred that the specific expression of hybrids is positively related to drought resistance heterosis and the over-dominance effect is the main genetic effect of the drought-resistant heterosis of *Zhangza 19*.

Heterosis results from polygenic interactions. Although it is difficult to attribute heterosis to one or several genes, the accumulation of the single-gene effects is important in the formation of heterosis [30]. The current analysis showed that there were 18 drought-resistant and heterotic genes; expression of *SiMYBS3* was significantly upregulated in *Zhangza 19* and showed a strong positive correlation with other genes, indicating that it might have some synergistic regulatory effect.

### 3.2. Functions of SiMYBS3

*MYB* genes are involved in plant growth and development. For example, overexpression of *Arabidopsis MYBH* (*At5g47390*) promoted the elongation of the hypocotyl [31], and its expression was induced by ABA, GA, CdCl_2_, and NaCl [32]. Overexpression of *MYB56* in *Arabidopsis* produced larger seeds [33]; overexpression of *MYB70* in *Arabidopsis* increased sensitivity of germination in response to exogenous ABA [34]; overexpression of R2R3-MYB TF *OsMYBAS1* promoted seed germination of rice at different sowing depths [35]; and rice R1-MYB TF *MORE FLORET1* regulated rice spikelet development [36]. In this study, overexpression of *SiMYBS3* led to earlier flowering in *Arabidopsis*, indicating that the *SiMYBS3* gene is involved in plant growth and development.

Some MYB genes in foxtail millet have been confirmed to be involved in stress response through transgenic overexpression. For example, overexpression of the foxtail millet *SiMYB3* gene enhanced tolerance to low-nitrogen stress in rice [37]. Overexpression of *SiMYB19* in rice enhanced salt resistance [38] and overexpression of *SiMYB56* conferred drought tolerance [39]. In our study, drought-induced upregulation of *SiMYBS3* expression in *Zhangza 19* and overexpression of *SiMYBS3* in *Arabidopsis* conferred drought tolerance. *SiMYBS3*-overexpression lines also showed stronger ABA tolerance than the WT and *mybs3* mutant. *SiMYBS3* contained the *cis*-acting element ABRE, a drought-responsive element in the promoter region, indicating that *SiMYBS3* may respond to drought stress through an ABA-dependent signal transduction pathway.

Our results showed that *SiMYBS3* regulates plant growth and development and is involved in drought tolerance, suggesting that overexpression of *SiMYBS3* provides a “stress signal” to the plant, causing a “stressful state”, prompting an earlier shift from vegetative growth to reproductive growth, hence flowering and fruiting earlier, and thereby reducing the damage caused by stress. Our results suggest that *SiMYBS3* identified from *Zhangza 19* can be used as candidate genes for molecular breeding of drought resistance in crops, especially in foxtail millet and wheat.

## 4. Materials and Methods

### 4.1. Plant Materials and Growth Conditions

The *Setaria italica* hybrid cultivar “*Zhangza 19*” and its female parent A2 and male parent DH2 were used in this study. *Zhangza 19* and its parents A2 and DH2 were obtained from the Zhangjiakou Academy of Agricultural Sciences of Hebei Province, China. Seeds were sown in a nutrition matrix and grown in an artificial climate chamber (23 °C, humidity of 65%, 16/8 h = day/night). Three-week-old seedlings were subjected to natural drought treatments (without watering). Leaf samples were collected on the 9th day for RNA extraction when the soil moisture content reached 20%. Each sample contained three independent biological replicates.

Tobacco (*Nicotiana tabacum*) and *Arabidopsis thaliana* strain Col-0 grown in the same environment as *S. italica* were used for subcellular localization and transformation, respectively. Seed of *Arabidopsis* T-DNA insertion mutant *mybs3* (*SAIL_205_B08C1*) was obtained from AraShare (http://www.arashare.cn/index/, accessed on 24 August 2022) and plants were genotyped by PCR and RT-PCR. The washing and planting methods for *Arabidopsis* were described earlier [40].

### 4.2. RNA Extraction, cDNA Synthesis, and RNA-Seq

Total RNA was isolated from 18 harvests (2 treatments × 3 genotypes × 3 biological replicates) using an RNA extraction kit (Coolaber, Beijing, China). The quantification and quality of the total RNA were assessed using an Agilent 2100 Bioanalyzer (Agilent, Santa Clara, CA, USA). High-quality RNA was prepared to construct a cDNA library. The mRNA was captured using magnetic oligo (dT) beads and fragmented using heat and magnesium. The first strand cDNA was synthesized using a random primer (Biomed, Beijing, China), and the second strand cDNA was synthesized with dNTPs, RNase H, and DNA polymerase. After repairing, A-tailing, adapter ligation, PCR amplifying, and the products were purified to generate the final libraries. The BGISEQ-500 platform (BGI, Shenzhen, China) was used for library sequencing. 

The genomic data of *Setaria italica* were downloaded from Phytozome V13 (https://phytozome-next.jgi.doe.gov/, accessed on 9 May 2021). The fastp-0.20.0 program [41] was used to trim adapters and low-quality sequences. Among the 855,140,406 raw reads produced, 821,298,598 were clean reads, and the clean read generation rate was higher than 96%. The cleaned FASTQ files were mapped to the reference genome to generate BAM files by STAR V2.7.9a [42] software, with the parameters (--readFilesCommand zcat, --runThreadN 20, --outSAMtype BAM), and sorted by using Samtools v1.12 [43]. Counts were counted by the featureCounts program of Subread v2.0.1 [44], and then FPKM quantification was performed using R language with the formula FPKM = total exon Fragments/(mapped reads(Millions) × exon length (KB)). Volcano plots were drawn using ggplot2 software [45]. The raw data were submitted to the NCBI database under accession number: PRJNA918739.

### 4.3. WGCNA Analysis and Identification of DEGs

WGCNA, an *R* package for weighted correlation network analysis, was performed on the DEG expression matrix to mine the key modules related to drought response. DEGs were filtered using the DESeq2 algorithm with an expression of |log_2_ Fold Change |> and a threshold of *p* < 0.05 [46]. DEGs of parents and hybrids were screened according to the FPKM value and log_2_ Fold Change, and the screening criteria for heterotic genes were as follows: the intersection of Fnodiff_FPKM > 1, Mnodiff_FPKM > 1, and F1diff_FPKM > 50 and the intersection of 1 < F_log2FC < 2, 1 < M_log2FC < 2, and F1_log2FC > 3. Correlations between genes were analyzed using the R package ggcorrplot, and clustering was performed by the “ward. D2” method.

### 4.4. Cis-Acting Elements and Phylogenetic Tree Analysis

PlantCARE [47] was used to predict the *cis*-elements in upstream 2 kb genomic DNA sequences of genes. The locations of *cis*-elements in the gene promoters were visualized using TBtools [48]. *SiMYBS3* gene homologous in different plant species were identified using the NCBI-BLASTP program, only one hit was obtained for the neighbor-joining (NJ) tree construction and the tree was visualized using MEGA [49].

### 4.5. Real-Time Quantitative PCR

Real-time quantitative (qRT) PCR was performed in triplicate using a ChamQ Universal SYBR qPCR Master Mix (Vazyme, Nanjing, China). Data collection and analyses were conducted using a CFX96 system (Bio-Rad, Hercules, CA, USA). The relative gene expression levels were calculated by the 2^−∆∆CT^ method. Primers used for PCR are listed in the Supplementary Materials, Table S1.

### 4.6. Subcellular Localization and Transcriptional Activity Analysis

*SiMYBS3* was amplified by PCR using primers *SiMYBS3*-F2 and *SiMYBS3*-R2 and subsequently subcloned into the pBWA(V)HS-GLosgfp expression vector. The recombinant plasmid was transferred into GV3101 and then transformed into tobacco leaves for subcellular localization using a Nikon C2-ER confocal microscope (Nikon, Tokyo, Japan).

The coding regions were cloned into the pGBKT7 vector for yeast autoactivation assays using primers *SiMYBS3*-F3 and *SiMYBS3*-R3. To assess the reliability of the autoactivation, different concentrations of the surviving clones grown on a SD/-Trp medium were dotted on SD/-Trp/-His medium. The resulting yeast growth was photographed after incubation at 30 °C for 3 d. Primer information is listed in the Supplementary Materials, Table S1.

### 4.7. Phenotypic Observations in Arabidopsis

Homozygous *Arabidopsis* transformants were produced using the floral dip method [50] and positive lines were identified as described in a previous study [40]. Seeds of wild-type *Arabidopsis* Col-0, third-generation overexpression lines, and *mybs3* mutants were spotted on plates containing ½ MS, 200 mM mannitol, and 1 µM abscisic acid (ABA) for germination experiments. Average germination rates were recorded after 3 d of vernalization at 4 °C and 3 d of normal light. To further investigate the drought tolerance of transgenic *Arabidopsis*, 15-day-old seedlings were subjected to natural drought stress (without watering) for one week and re-watered after drought stress, and survival rates were recorded 3 d after re-watering.

### 4.8. Statistical Analysis

Microsoft Excel 2010 was used for data analysis and plotting. Values are shown as mean ± SD (standard deviation) and significant differences are indicated with “*” (*p* < 0.05) or “**” (*p* < 0.01).

## Data Availability

Data supporting the discovering of our work are available within the paper and its Supplementary Materials.

## Supplementary Materials

The following supporting information can be downloaded at: https://www.mdpi.com/article/10.3390/ijms24065418/s1.
