# Supplementary material for: SiMYBS3, Encoding a Setaria italica Heterosis-Related MYB Transcription Factor, Confers Drought Tolerance in Arabidopsis
Source: Int J Mol Sci. 2023 Mar 12;24(6):5418. doi: 10.3390/ijms24065418 (PMC10049516; doi:10.3390/ijms24065418)
Supplement: Supplementary file 1 [file ijms-24-05418-s001.zip › Table S1.pdf]

Table S1. Primer pairs used in the experiment

Primer	Sequence	Application
SiMYBS3- F1	5´- ATGACGCGGCGGTGCTCGCACTGCA -3´	Gene cloning
SiMYBS3- R1	5´- TCAGGCCTGAGCCCTTGTTTGAGGATTT -3´	
SiMYBS3- F2	5´-ATTTGGAGAGAACACGGGGGACTTTGCAACATGACGCGGCGGTGCTCGCACTGCA-3'	Subcellular localization
SiMYBS3- R2	5´-GGAACCACTCCCTGAAGCGGCCGCTGTACAGGCCTGAGCCCTTGTTTGAGGATTT-3'	
SiMYBS3- F3	5´-TCAGAGGAGGACCTGCATATGATGACGCGGCGGTGCTCGCACTGCA -3´	Transcriptional activity
SiMYBS3- R3	5´-TCGACGGATCCCCGGGAATTCTCAGGCCTGAGCCCTTGTTTGAGGATTT - 3´	
SiMYBS3- F4	5´-TTCCGCTGTTGCAGAGAGTC - 3´	Quantitative real-time PCR
SiMYBS3- R4	5´-TCGACATGCCCACAAGTTCATC -3´	
SiActin- F	5´-CGCATATGTGGCTCTTGACT -3´	
SiActin- R	5´-GGGCACCTAAATCTCTCGC -3´	
