# Supplementary material for: SiMYBS3, Encoding a Setaria italica Heterosis-Related MYB Transcription Factor, Confers Drought Tolerance in Arabidopsis
Source: Int J Mol Sci. 2023 Mar 12;24(6):5418. doi: 10.3390/ijms24065418 (PMC10049516; doi:10.3390/ijms24065418)
Supplement: Supplementary file 1 [file ijms-24-05418-s001.zip › Table S2.pdf]

Table S2.

18 genes involved in the response to drought stress and heterosis

**Gene ID**	**Annotation description**
Seita.2G405500	Serine/Threonine - protein kinase
Seita.9G321800	Transcription factor MYBS3
Seita.5G308400	UDP-glucuronosyl and UDP-glucosyl transferase
Seita.9G516500	Ethylene-responsive transcription factor
Seita.3G126900	RNA polymerase sigma-70
Seita.3G136300	Protein CHUP1, chloroplastic
Seita.9G131700	DUF1336 domain-containing protein
Seita.9G391300	Cytochrome P450
Seita.4G072300	Phosphofructokinase domain
Seita.3G375500	Adaptor protein Enigma and related PDZ-LIM proteins
Seita.5G231500	Serine/threonine - protein kinase
Seita.9G128600	dihydroceramidase (ACER3, YDC1)
Seita.5G177000	de-etiolated-1 (DET1)
Seita.5G340900	lysocardiolipin and lysophospholipid acyltransferase (LCLAT1, AGPAT8)
Seita.5G300000	Serine O-acetyltransferase
Seita.5G411200	BTB and MATH domain -containing protein 42
Seita.5G330600	E3 ubiquitin-protein ligase RNF5
Seita.5G408100	Expressed protein
