# Supplementary figures and images for: SiMYBS3, Encoding a Setaria italica Heterosis-Related MYB Transcription Factor, Confers Drought Tolerance in Arabidopsis
Source: Int J Mol Sci. 2023 Mar 12;24(6):5418. doi: 10.3390/ijms24065418 (PMC10049516; doi:10.3390/ijms24065418)

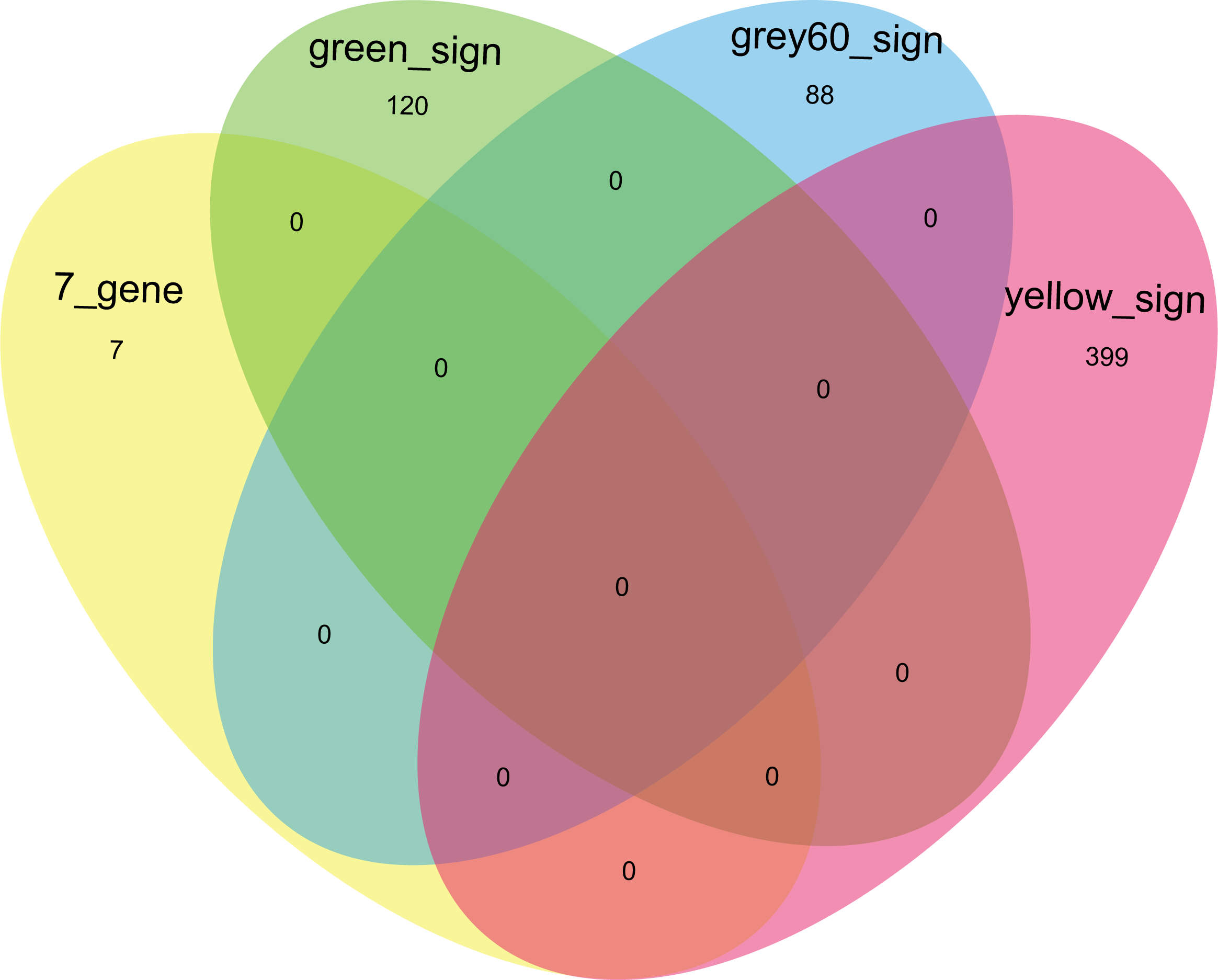

Supplement: Supplementary file 1 [file ijms-24-05418-s001.zip › Figure S1.tif]
